# Expression Pattern and Prognostic Value of Key Regulators for m6A RNA Modification in Hepatocellular Carcinoma

**DOI:** 10.3389/fmed.2020.00556

**Published:** 2020-09-16

**Authors:** Lele Zhang, Yiting Qiao, Jiacheng Huang, Dalong Wan, Lin Zhou, Shengzhang Lin, Shusen Zheng

**Affiliations:** ^1^The First Affiliated Hospital, Zhejiang University School of Medicine, Division of Hepatobiliary and Pancreatic Surgery, Department of Surgery, Hangzhou, China; ^2^School of Medicine, Zhejiang University, Hangzhou, China; ^3^NHC Key Laboratory of Combined Multi-organ Transplantation, Hangzhou, China; ^4^Key Laboratory of the Diagnosis and Treatment of Organ Transplantation, Research Unit of Collaborative Diagnosis and Treatment for Hepatobiliary and Pancreatic Cancer, Chinese Academy of Medical Sciences (2019RU019), Hangzhou, China; ^5^Key Laboratory of Organ Transplantation, Hangzhou, China; ^6^Shulan (Hangzhou) Hospital Affiliated to Zhejiang Shuren University Shulan International Medical College, Hangzhou, China

**Keywords:** m6A, hepatocellular carcinoma, bioinformatics, prognostic signature, nomogram

## Abstract

As the most prevalent type of mRNA modification in mammals, N6-methyladenosine (m6A) is involved in various biological processes. Accumulating studies have indicated that the deregulation of m6A RNA modification is linked to cancer and other diseases. However, its implications in hepatocellular carcinoma (HCC) remain poorly characterized. Herein, we sought to investigate the expression pattern of 13 key regulators for m6A RNA modification and to evaluate their prognostic value in HCC. First, we systematically analyzed data from The Cancer Genome Atlas (TCGA) database pertaining to patient clinical information and mRNA gene expression data. We found that 11 out of 13 key regulators for m6A RNA modification showed significantly higher expression levels in HCC. Subsequently, we identified two subgroups (clusters 1 and 2) *via* consensus clustering based on the expression of 13 m6A RNA methylation regulators. Cluster 2 had a worse prognosis and was also significantly correlated with higher histological grade and pathological stage when compared with cluster 1. Moreover, cluster 2 was remarkedly enriched for cancer-related pathways. We further constructed a robust risk signature of five regulators for m6A RNA modification. Further analysis indicated that this risk signature could be an independent prognostic factor for HCC, and the prognostic relevance of this five-gene risk signature was successfully validated using the Gene Expression Omnibus (GEO) dataset. Finally, we established a novel prognostic nomogram on the basis of age, gender, histological grade, pathological stage, and risk score to precisely predict the prognosis of patients with HCC. In summary, we herein uncovered the vital role of regulators for m6A RNA modification in HCC and developed a risk signature as a promising prognostic marker in HCC patients.

## Introduction

Hepatocellular carcinoma (HCC) remains among the most prevalent and deadly types of cancer worldwide, with more than 700,000 deaths documented annually (1). Epidemiological studies have shown that hepatitis virus infection, alcohol abuse, and aflatoxin contamination are primary risk factors for HCC (2). Due to the lack of apparent symptoms at the early stages of HCC, the majority of patients diagnosed with this cancer are first identified in an advanced stage where the complication of intrahepatic and/or extrahepatic metastasis has taken place (3). Even though the prognosis for HCC patients has improved due to recent advances in various treatment approaches, including surgical tumor resection, targeted drug therapy, transarterial chemoembolization, and liver transplantation, the 5-year survival rate remains dismal due to the high rate of metastasis and recurrence (4). At present, the tumor, lymph node, and metastasis (TNM) staging system is still the most widely adopted prognostic indicator for monitoring the progress of HCC. However, HCC is highly heterogeneous; therefore, patients with the same TNM stage often present remarkable differences in survival outcomes and treatment responses. Therefore, to improve the unsatisfactory outcomes of patients with HCC, it is important to identify novel and reliable molecular signatures for prognostic prediction.

Methylation at the position N6 of adenosine, also known as N6-methyladenosine (m6A) modification, is evolutionarily conserved and widely present in most eukaryotic species (yeast, plants, and mammals) and viral mRNA (5, 6). The process of m6A modification is reversible and dynamic and is controlled by methyltransferases (“writers”), demethylases (“erasers”), and methyl-binding proteins (“readers”) (7). Methyltransferases, such as METTL3, RBM15, KIAA1429, METTL14, WTAP, and ZC3H13 are responsible for the methylation modification of RNA (8). Demethylases, including ALKBH5 and FTO, mediate the process of demethylation of RNA (9, 10). Methyl-binding proteins, including YTHDF1, HNRNPC, YTHDC1, YTHDF2, and YTHDC2, can recognize m6A-modified sites and preferentially bind to such sites to regulate downstream signals (11). RNA m6A modification is involved in many vital cellular processes, such as gene expression, alternative splicing, degradation, translation of mRNA, and RNA–protein interaction (12).

The poor prognosis of cancer patients is due to the unique malignant biological characteristics of the cancer, including epithelial–mesenchymal transition (EMT), cancer stem cell formation, signaling transduction, tumor angiogenesis, and cancer metabolism. Several studies have shown that aberrant m6A RNA modification plays key roles in these biological processes closely associated with the HCC progression by regulating mRNA stability or protein translation (13–15). For example, METTL3- and YTHDF1-dependent m6A modification could promote EMT by enhancing the translation of Snail mRNA in liver cancer (16). In hypoxic environment, the expression of ALKBH5 was stimulated in a HIF-dependent manner in breast cancer. Overexpression of ALKBH5 decreased m6A modification and stabilizes NANOG mRNA, thus resulting in higher stemness (17). Considering that hypoxia also plays an important role in the progression of HCC, m6A may also promote the formation of cancer stem cells through a similar approach. As research continues, m6A modification has been shown to participate in the many signaling pathways, including but not limited to the MYC/CEBPA, Wnt/PI3K-Akt, AFF4/NF-κB/MYC, YAP, and TGF-β signaling pathways, to promote cancerous growth as well as angiogenesis (18, 19). In addition, m6A modification can modulate cancer metabolism through downregulating the translation of ATG5/7, the key signal node for autophagy, as well as upregulating the translation of 6PGD, the central player of pentose phosphate pathway (20, 21). To summarize, deregulation of m6A modification profoundly promoted the malignancy of cancer. This explains why m6A RNA modification has prognostic impacts on patients with HCC.

In this study, transcriptome data from The Cancer Genome Atlas (TCGA) datasets were used to assess the expression of 13 key regulators for m6A RNA modification in HCC. Additionally, HCC patients were categorized into two clusters according to the expression pattern of regulators for m6A RNA modification by consensus clustering, and two clusters exhibited significantly different clinical outcomes. Furthermore, a risk signature prognostic prediction model was established and showed a favorable predictive value for HCC patients. More importantly, the prognostic relevance of this risk signature was successfully validated in the Gene Expression Omnibus (GEO) database.

## Materials and Methods

### Data Collection

RNA-sequencing transcriptomic data and corresponding clinical information for patients with HCC were obtained from TCGA (https://portal.gdc.cancer.gov/; until February 21, 2020). A total of 374 HCC cases and 50 normal adjacent tumor tissues were included for further analysis.

Thirteen currently known genes, including YTHDF1, YTHDF2, YTHDC1, YTHDC2, METTL3, METTL14, ALKBH5, FTO, HNRNPC, KIAA1429, RBM15, WTAP, and ZC3H13, are recognized as m6A methylation regulators. The expression data of these 13 genes were extracted for subsequent analysis from the HCC cohort of the TCGA database. For external validation, we used an independent cohort (GSE54236) containing 78 HCC samples with corresponding gene expression data and the survival information, which were obtained from the GEO (http://www.ncbi.nlm.nih.gov/geo).

### Bioinformatics Analysis

Differentially expressed genes encoding m6A RNA methylation regulators between HCC and normal tissues were screened using the Wilcoxon test method in R (version R 3.6.3, https://www.r-project.org/). Significance criteria were as follows: false discovery rate (FDR) < 0.05 and absolute log2 fold change (FC) > 1. Subsequently, a vioplot was used to exhibit the expression of these m6A-related genes in 374 HCC patients and 50 normal adjacent samples. Spearman correlation analyses were conducted using R to identify the association between m6A RNA methylation regulators.

To assess the link between m6A RNA methylation regulators expression and HCC prognosis, HCC cohort was clustered into two different subgroups using the “ConsensusClusterPlus” R package. Principal component analysis (PCA) was then carried out using the “ggplot2” and “limma” package to verify the results of the classification. A survival curve was plotted to compare survival between subgroups based on the Kaplan–Meier analysis log-rank test. The difference in clinical parameters between the two clusters was determined using the Chi-square test. In order to conduct functional annotation of the genes with different expression in two subgroups, we performed Gene ontology (GO) and Kyoto Encyclopedia of Genes and Genomes (KEGG) analyses.

Univariate Cox regression analyses were utilized to assess the relationship between m6A-related genes and overall survival (OS). Subsequently, to avoid overfitting, we performed least absolute shrinkage and selection operator (LASSO) Cox regression to eliminate the highly correlated genes with the “glment” package. Ultimately, a five-m6A-regulatory-gene risk signature was identified. To generate a risk score, we multiplied the gene expression and its coefficient obtained from the LASSO Cox regression. Median risk scores were then used to separate HCC patients into low- and high-risk groups. Kaplan–Meier analysis was performed using “survival” package. Receiver operating characteristic (ROC) curves were used to examine the accuracy of the model for prognostic prediction. The differences in clinicopathological variables between low- and high-risk groups were assessed *via* Chi-square text and visualized *via* a heatmap. In addition, univariate and multivariate Cox regression analyses were used as a means of assessing whether the risk score was an independent prognostic indicator.

To validate the prognostic value of this five-m6A-regulatory-gene risk signature, we used GSE54236 datasets as the validation cohort. Patient risk scores were calculated using the same formula as above. We applied the same cutoff criteria to classify the patients into low- and high-risk groups. Subsequently, Kaplan–Meier survival analysis and ROC curve analysis were performed to assess the prognostic value.

Finally, clinical factors (gender, age, histologic grade, and pathological stage) and risk score were utilized to develop a prognostic nomogram to predict 1-, 3-, and 5-year survival of patients with HCC *via* “rms” package.

All R packages mentioned above were obtained from http://www.bioconductor.org.

### Statistical Analysis

R software (version 3.6.3) was utilized for all statistical analyses, and *p* < 0.05 was the significance threshold.

## Results

### Identification of Differentially Expressed m6A RNA Modification Regulators in HCC

**Figure 1:**
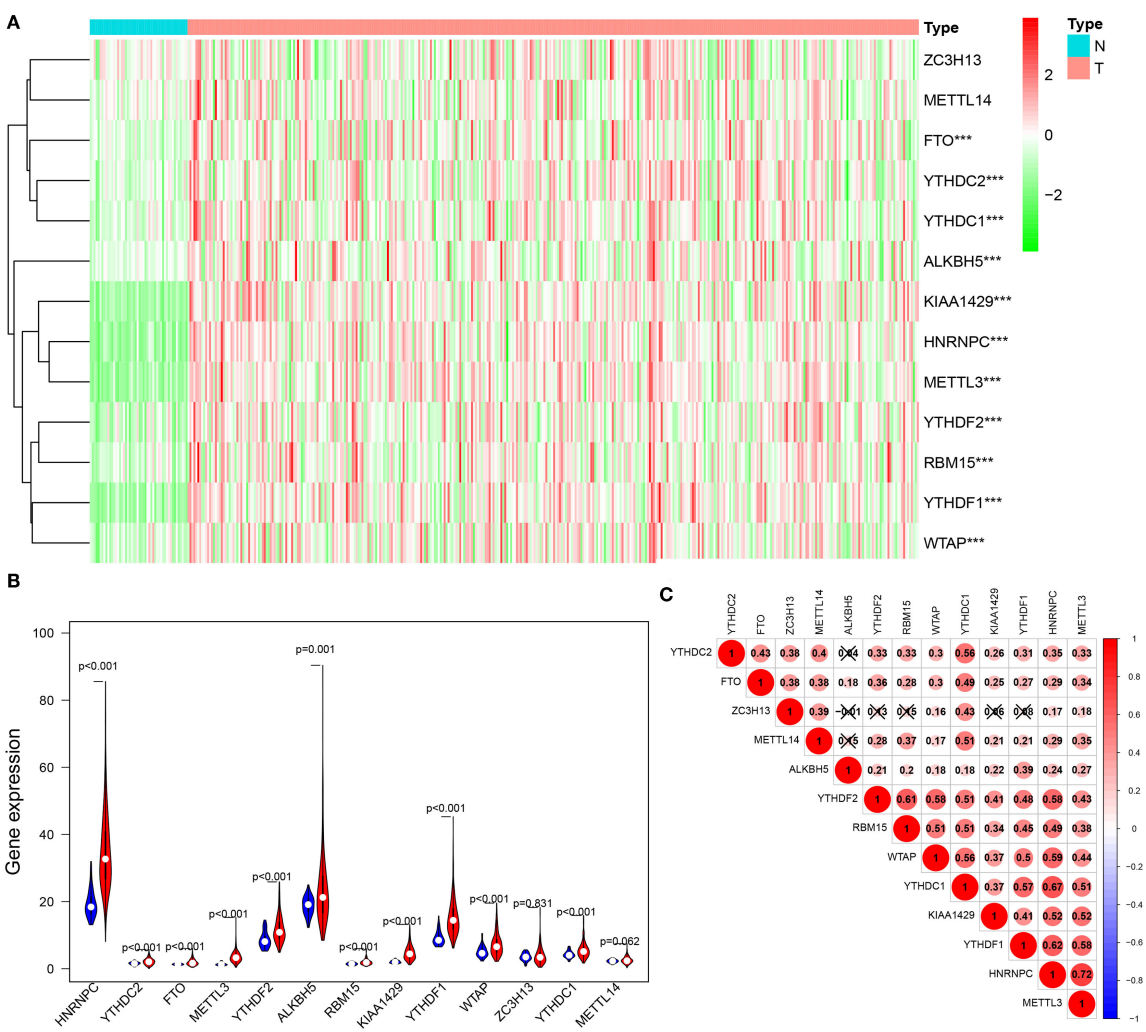
Expression of m6A modification regulators in HCC. **(A)** The heatmap visualizes the expression levels of m6A RNA modification regulators in each sample. “N” represents normal samples and “T” represents tumor samples. Green represents low expression and red represent high expression. **(B)** The vioplot shows the differentially m6A RNA modification regulators in HCC. Blue represents normal sample and red represents HCC sample. White spot represents the median value of expression. **(C)** Spearman correlation analysis of the 13 m6A RNA modification regulators in HCC. ***p < 0.001.

We conducted a differential expression analysis of 13 m6A regulatory genes in HCC (*n* = 375) and adjacent tissues (*n* = 50). Heatmap clearly revealed that most of these m6A-related genes were differentially expressed between HCC and control tissues (Figure 1A). Specifically, the expression levels of HNRNPC, YTHDF2, FTO, METTL3, YTHDF2, ALKBH5, RBM15, KIAA1429, YTHDF1, WTAP, and YTHDC1 (all *p* < 0.001) were remarkably higher in tumor samples than those in normal tissues. There was no significant difference for ZC3H13 (*p* = 0.831) and METTL14 (*p* = 0.062) (Figure 1B). Moreover, a correlation analysis was performed to further understand the intrinsic association between 13 m6A RNA modification regulators. Figure 1C shows that the correlation between METTL13 and HNRNPC is the most significant. The HNRNPC expression level is most likely to be positively correlated with METTL13.

### Use of Consensus Clustering Based on m6A RNA Modification Regulators to Identify Two HCC Patient Clusters With Distinct Clinical Outcomes

To further investigate the clinical relevance of 13 m6A RNA modification regulators, we clustered HCC patients into subgroups according to their gene expression patterns.

**Figure 2:**
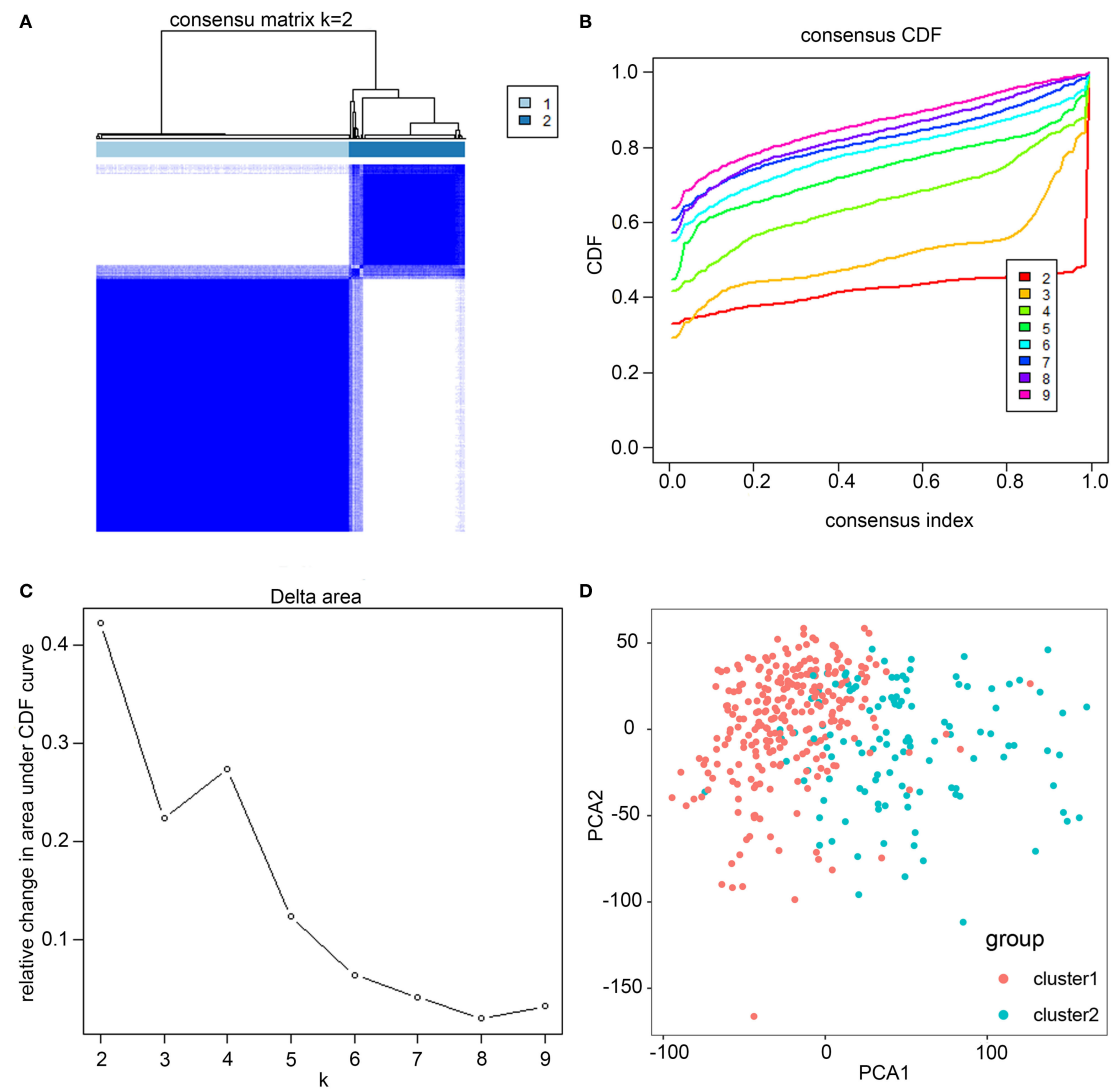
Consistent cluster analysis of HCC. **(A)** The correlation between subgroups when cluster numbers *k* = 2. **(B)** Cumulative distribution function (CDF) is displayed for *k* = 2–9. **(C)** The relative change in area under the CDF curve for *k* = 2–9. **(D)** Principal component analysis of the RNA-seq data. Red dots represent cluster 1 and cyan dots represent cluster 2.

**Figure 3:**
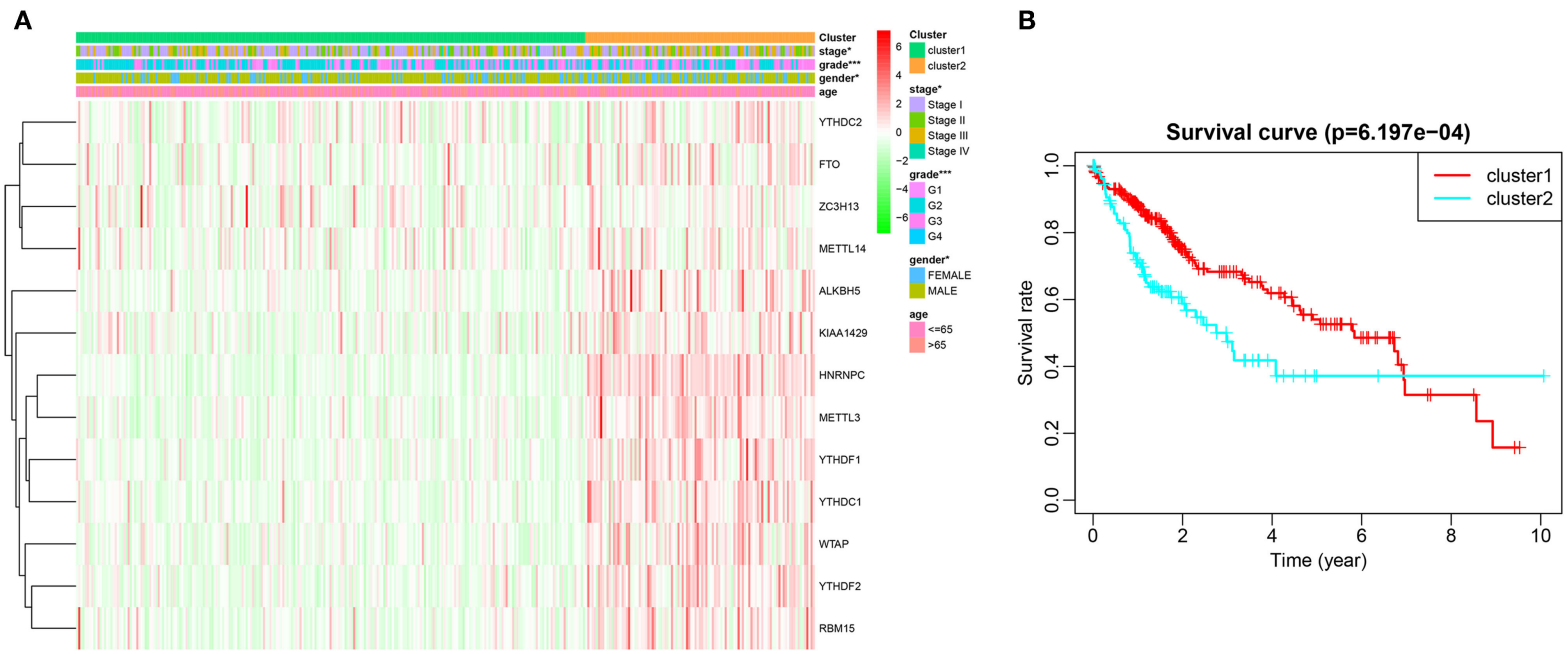
Difference in clinicopathological features and overall survival between cluster 1 and cluster 2. **(A)** Heatmap and clinicopathological characteristics of these two clusters. Green represents low expression and red represent high expression. **(B)** Comparison of overall survival (OS) between cluster 1 and cluster 2. *p < 0.05, ***p < 0.001.

Based on similarities in m6A RNA modification regulators, *k* = 2 gave the optimum clustering and the HCC cohort could be divided into two distinct and non-overlapping clusters (Figures 2A–C). In order to verify the result of the clustering, we further analyzed these two clusters by PCA. The PCA plot showed significant distinction between cluster 1 and cluster 2 (Figure 2D). We then assessed whether there were significant differences in OS and clinical parameters between these two clusters. As a result, a significantly better OS was observed in cluster 1, compared to that in cluster 2 (*p* < 0.01) (Figure 3B). Moreover, the expression level of most m6A RNA modification regulators of cluster 2 was higher than that of cluster 1 (Figure 3A). Compared with cluster 1, cluster 2 was significantly associated with female gender (*p* < 0.05), higher histologic grade (*p* < 0.001), and higher pathological stage (*p* < 0.05). No significant difference was observed for age (Figure 3A). Thus, the results of consensus clustering suggested a close association between the expression pattern of m6A RNA modification regulators and HCC malignancy.

**Figure 4:**
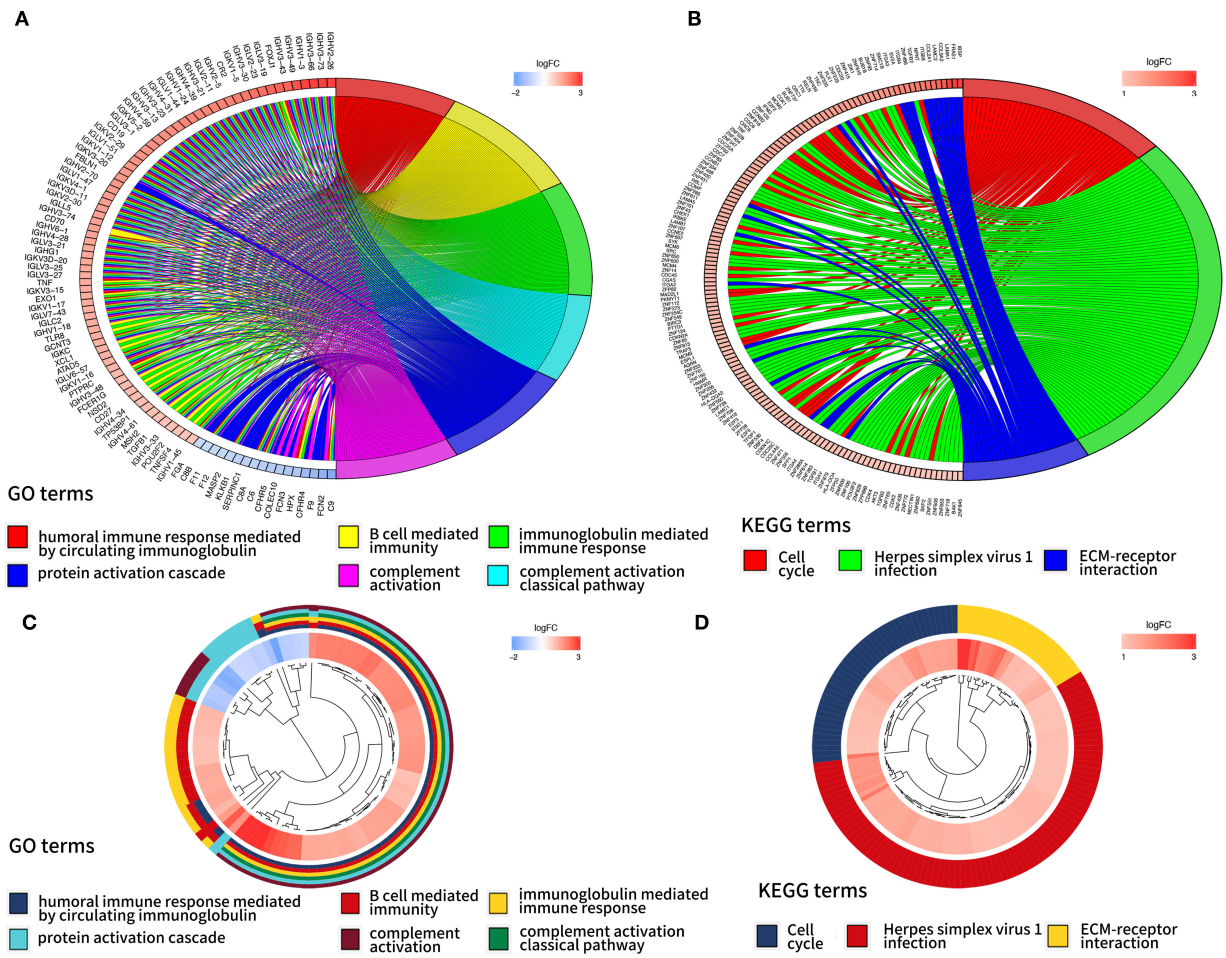
Gene ontology (GO) and Kyoto Encyclopedia of Genes and Genomes (KEEG) analyses of differentially expressed genes between two clusters. Function annotation on higher expressed genes in cluster 2 using GO terms **(A,B)** and KEGG pathway **(C,D)**.

In order to further interpret the clustering results from the perspective of fundamental biological processes, we performed GO and KEGG analyses on genes that are differentially expressed between cluster 1 and cluster 2. According to the results of the GO analysis, upregulated genes were primarily enriched in malignancy-related biological processes, such as “humoral immune response mediated by circulating immunoglobulin,” “B cell mediated immunity,” “immunoglobulin mediated immune response,” “complement activation, classical pathway,” and “protein activation cascade” (Figures 4A,B). The results of the KEGG analysis showed that these upregulated genes were significantly enriched in “cell cycle,” “herpes simplex virus 1 infection,” and “extracellular matrix (ECM)–receptor interaction” (Figures 4C,D).

### Establishment of a Prognostic Risk Model Based on the Expression Level of m6A Regulator Genes

**Figure 5:**
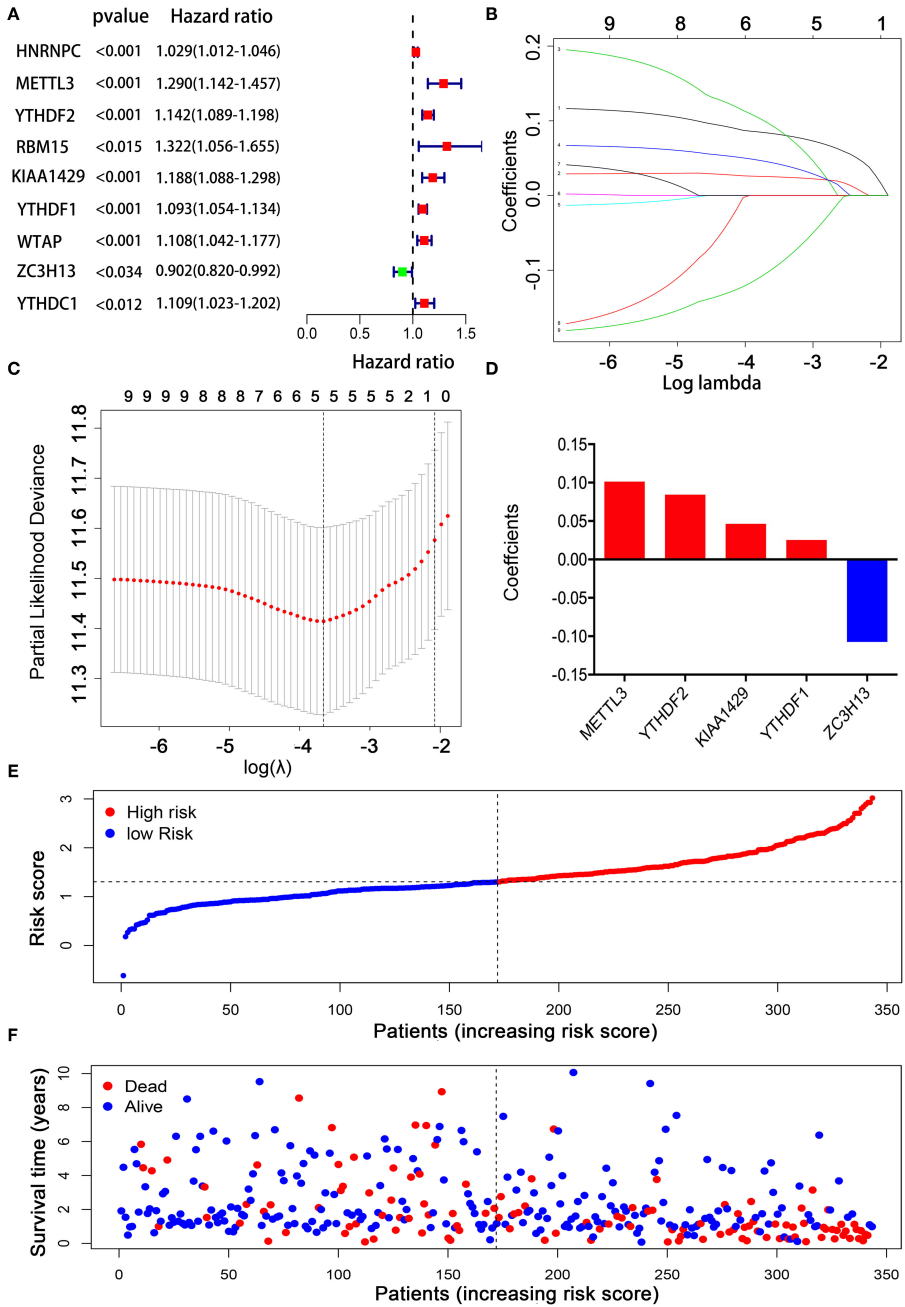
Establishment of the prognostic risk model based on m6A RNA modification regulator genes. **(A)** Univariate Cox regression analysis of the m6A RNA methylation regulators. **(B–D)** The process of constructing the signature using absolute shrinkage and selection operator (LASSO) Cox regression. **(E)** The distributions of risk scores in the prognostic model. **(F)** The distributions of survival status in the prognostic model.

Considering the strong association between m6A RNA methylation regulators and the prognosis of HCC patients, we applied a univariate Cox regression analysis on the expression levels of 13 key regulators. The results showed that nine out of 13 regulators were significantly correlated with OS (*p* < 0.05) (Figure 5A). Among these nine regulators, YTHDF1, YTHDF2, METTL3, KIAA1429, HNRNPC, WTAP, YTHDC1, and RBM15 were considered as risky genes, with HR > 1; meanwhile only ZC3H13 was considered as a protective gene, with HR < 1. Subsequently, LASSO Cox regression analysis was used to identify the m6A RNA modification regulators with the strongest prognostic power (Figures 5B,C). Ultimately, five optimal genes (YTHDF1, YTDFH2, METTL3, KIAA1429, and ZC3H13) were selected for the establishment of the risk model for HCC, and the corresponding coefficients from the LASSO algorithm (Figure 5D). The formula for calculating the risk score was as follows: risk score = (0.084 ^*^ expression value of YTHDF2) + (0.025 ^*^ expression value of YTHDF1) + (0.101 ^*^ expression value of METTL3) + (0.046 ^*^ expression value of KIAA1429) – (0.107 ^*^ expression value of ZC3H13).

**Figure 6:**
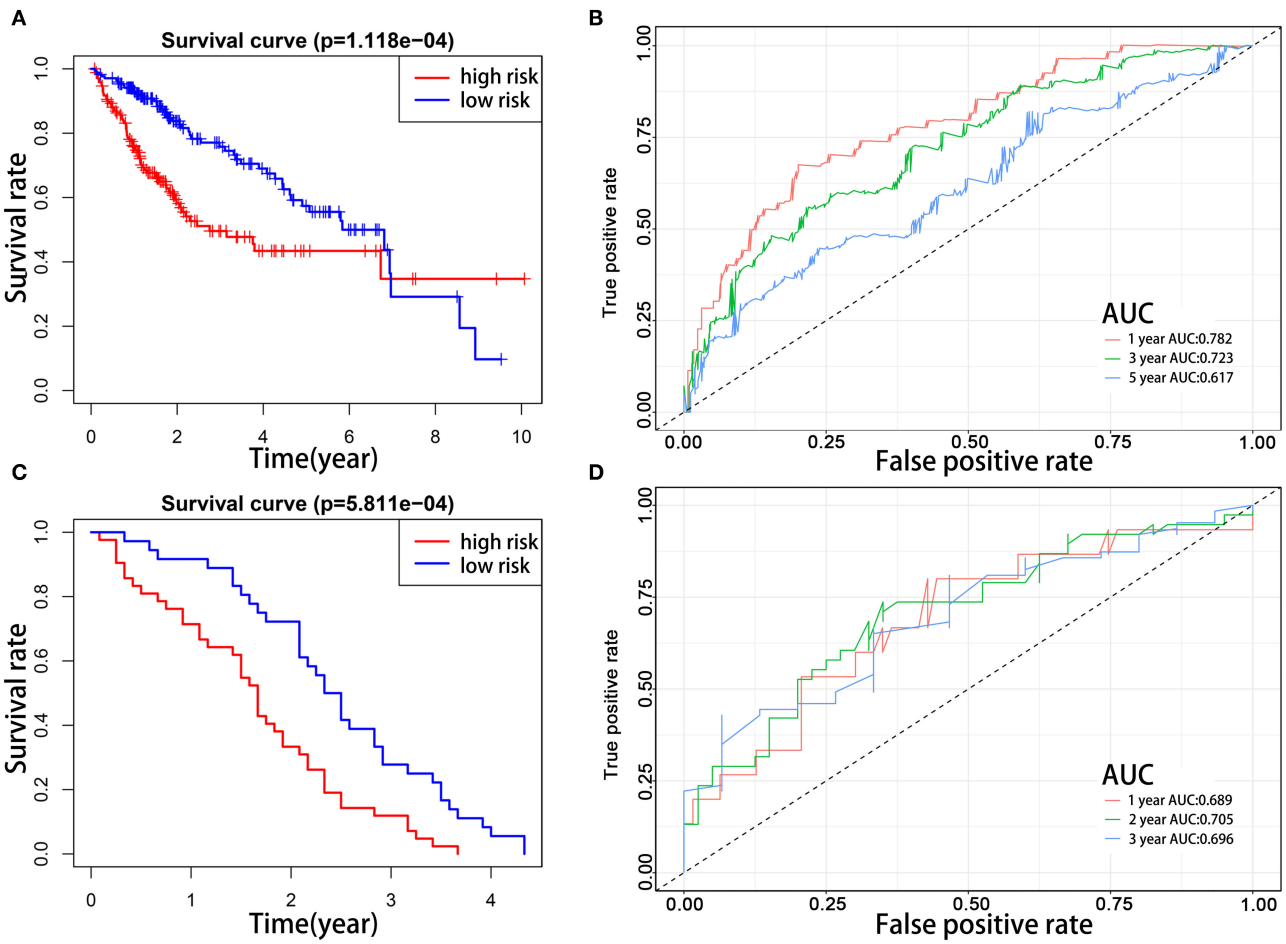
Kaplan–Meier survival analyses of prognostic model. Patients in two datasets were assigned to low-risk (blue) and high-risk (red) groups using median risk score as the cutoff. **(A,B)** In the TCGA cohort, the survival probability of the low-risk group is higher than the high-risk group (*p* = 1.118e−4). The 1-, 3-, and 5-year AUCs were 0.782, 0.723, and 0.617, respectively. **(C,D)** The prognostic model was validated in the GEO cohort. The survival probabilities were higher for the low-risk group than the high-risk group (*p* = 5.811e−04). The 1-, 2-, and 3-year AUCs were 0.689, 0.705, and 0.696, respectively.

**Figure 7:**
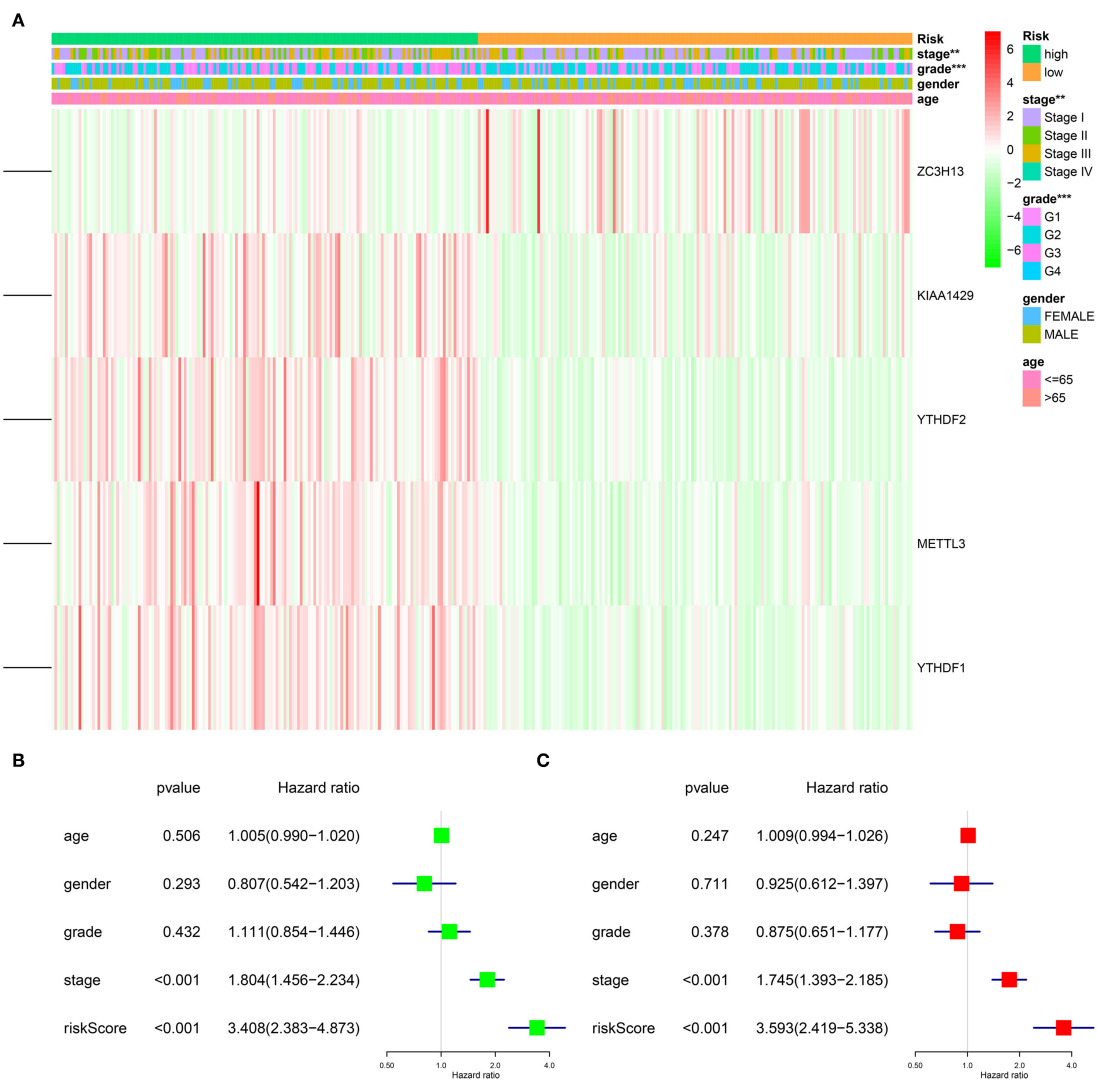
Impact of risk score and clinicopathological features on the prognosis of HCC patients. **(A)** Heatmap shows the distribution of clinicopathological features and the expression of five m6A RNA modification regulators in high- and low-risk groups. **(B)** Univariate Cox regression analyses of clinicopathological parameters and OS. **(C)** Multivariate Cox regression analyses of clinicopathological parameters and OS. **p < 0.01, ***p < 0.001.

To explore the prognostic role of this five-gene signature model, HCC patients were separated into low- and high-risk groups based on the median risk score. Survival analysis demonstrated a worse OS in patients with a high-risk score relative to patients with a low-risk score (Figure 6A, *p* = 1.118e−04). The 5-year OS rate was 43.4% in the high-risk group and 57.4% in the low-risk group. We then performed a ROC curve analysis and assessed the area under this curve (AUC) of 0.782, 0.723, and 0.617 for the 1-, 3-, and 5-year OS, respectively, which showed good predictive power for survival outcomes (Figure 6B). Moreover, the risk score distribution of patients with HCC was plotted, as shown in Figure 5E. A dot pot was used to display the survival status of each patient (Figure 5F). The expression of five prognostic genes in the high- and low-risk groups was displayed in a heatmap (Figure 7A). Clinical relevance was simultaneously plotted above the heatmap. When comparing the clinical parameters between the low- and high-risk groups, significant differences were observed in terms of stage (*p* < 0.01) and grade (*p* < 0.001).

### Validation of the Prognostic Signature Using the GEO Database

To evaluate the prognostic value of the five-gene signature for survival prediction in other datasets, we used the GEO microarray data (GSE54236) for validation (22). A total of 78 HCC patients in the GSE54236 cohort were divided into high-risk (*n* = 42) and low-risk (*n* = 36) groups according to the cutoff value of the TCGA cohort. Same as the results in the TCGA cohort, the survival analysis demonstrated that HCC patients in the low-risk group had markedly better OS compared to high-risk patients (Figure 6C, p = 5.811e−4). The AUCs for 1-, 2-, and 3-year OS were 0.689, 0.705, and 0.696, respectively, indicating that this prognostic model could predict OS of HCC patients with a good accuracy (Figure 6D). Since there was no patient survival beyond 5 years, the 5-year ROC curve was not plotted.

### The Five-Gene Risk Signature Independently Predicts the Prognostic of HCC Patients

After excluding cases with incomplete clinical information, 339 cases were eligible for Cox regression analysis. Univariate analysis revealed that the five-gene risk score and stage were significantly related to the OS of patients with HCC (Figure 7B, *p* < 0.001). In order to evaluate whether the five-gene risk signature was independent from other clinicopathologic characteristics as a prognostic factor for HCC, we additionally conducted multivariate Cox regression analyses, which demonstrated that both risk score and stage were independently correlated with OS for patients with HCC (Figure 7C, *p* < 0.001). These results demonstrated that the five-gene risk signature was able to predict prognosis independently of gender, age, histological grade and pathological stage, indicating that this five-gene risk signature could serve as an independent prognostic factor for HCC.

### Establishment of a Prognostic Nomogram for HCC

**Figure 8:**
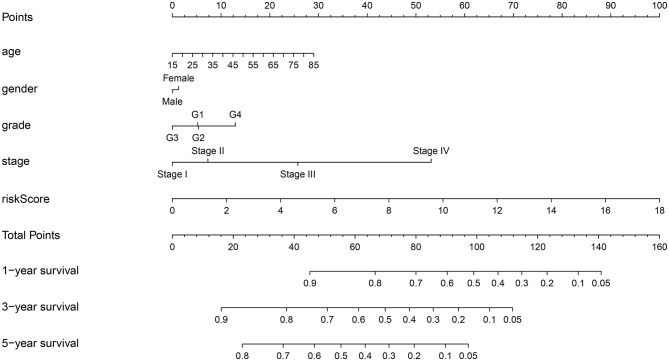
Establishment of prognostic nomogram by combining clinicopathologic characteristics and risk score.

To provide a quantitative method to predict the survival of individuals, we established a novel prognostic nomogram on the basis of age, gender, histological grade, pathological stage, and risk score (Figure 8). The results showed that the nomogram could systematically predict the 1-, 3-, and 5-year OS of patients with HCC.

## Discussion

Globally, HCC is the most prevalent type of liver malignancy, which ranks as the fourth of cancer mortality (1). However, there is currently no effective therapy, and the OS of patients with HCC is still far from satisfactory. Therefore, it is urgent to elucidate the underlying molecular mechanisms contributing to tumorigenesis in HCC. RNA m6A modification, as a new dimension of gene expression control, has aroused strong interest among the academic community in recent years. However, the study of m6A modification in the cancer field is still in its initial stage. Due to the wide application of RNA-seq and microarray techniques, risk scoring systems based on multiple-gene signature are increasingly frequently applied to predict prognosis for human cancers (23–25). In the present study, we established a prognostic signature using five m6A RNA modification regulators. Encouragingly, the risk score was able to independently predict the prognosis of HCC patients. Therefore, the risk signature in this study can help clinicians perform individualized survival predictions more accurately.

As shown in our prognostic model, ZC3H13 was the only m6A-related gene positively associated with HCC patient prognosis, indicating that ZC3H13 might exert a suppressive effect on HCC. ZC3H13 encodes a CCCH-type zinc finger protein, which plays an important role in the modulation of RNA m6A modification in the nucleus (26, 27). To date, even though a few studies have reported the correlation between ZC3H13 and human tumors, its biological function still needs further investigation. According to published studies, ZC3H13 exhibited heterogeneous roles in various types of human cancer. For example, Zhu et al. found that ZC3H13 inhibits colorectal cancer (CRC) proliferation and invasion *via* inactivating Ras-ERK signaling pathway, suggesting that ZC3H13 acts as a tumor suppressor in CRC (28). Similarly, Kim et al. demonstrated that ZC3H13 is often mutated in CRC, suggesting that ZC3H13 might function as a tumor suppressor (26). On the contrary, Gewurz et al. revealed that ZC3H13 acts as a key upstream regulator of the NFκB activation pathway. Since hyperactivation of the NFκB promotes tumor proliferation and invasion, ZC3H13 might function as an oncogene (29). Therefore, the specific role of ZC3H13 in HCC remains elusive, which requires in-depth research.

Our prognostic model showed that the expression of the remaining four genes (YTHDF1, YTHDF2, METTL3, and KIAA1429) was adversely associated with the prognosis of patients with HCC. Chen et al. reported that METTL3 is significantly upregulated in HCC. Overexpression of METTL3 in HCC is linked to poor prognosis. In addition, knockdown of METTL3 impairs HCC oncogenicity and lung metastasis, indicating that METTL3 might act as an oncogene in HCC. Mechanistically, METTL3 promotes the progression of HCC through post-transcriptional silencing SOCS2 (a tumor suppressor gene) in a YTHDF2-dependent manner (15). Vice versa, Lin et al. revealed that the suppression of METTL3 attenuated EMT through downregulating the translation of Snail (16). In agreement with the oncogenic functions of METTL3 in HCC, similar observations have recently been reported in several other types of cancer, namely, AML (30), GMB (31), bladder cancer (32), gastric cancer (33), and breast cancer (34).

KIAA1429, a relatively new component of the m6A “writer” complex, has also been reported to be upregulated in HCC (35). Clinically, overexpression of KIAA1429 is linked to a worse prognosis for HCC patients. Mechanistically, KIAA1429 promotes HCC progression by inhibiting ID2 *via* upregulating its m6A level (35). Similarly, Lan et al. also showed that KIAA1429 is highly expressed in HCC and correlated with poor prognosis of HCC patients. Their study demonstrated that silencing KIAA1429 inhibited proliferation and metastasis of cancer cells (36). Mechanistically, KIAA1429 could induce m6A modification of GATA3 pre-mRNA, resulting in the degradation of GATA3 pre-mRNA, thus contributing to HCC progression. Interestingly, Qian et al. reported that KIAA1429 could exert oncogenic functions in breast cancer through positive regulating CDK1 in an m6A-independent manner (37). These studies suggest that KIAA1429 is an oncogenic protein that functions through multiple signaling pathways.

YTHDF1 and YTHDF2 are both members of the YTH domain family, which also includes YTHDF3, YTHDC1, and YTHDC2. YTHDF1 is an m6A “reader,” which recognizes and binds to m^6^A-modified mRNA, thus improving the translation efficiency of their target RNAs (38). In a recent study, YTHDF1 was reported to be upregulated in HCC, and its overexpression is highly related to unfavorable prognosis (39), which is consistent with the observations of Zhao et al. (40). Additionally, it has been reported that YTHDF1 is highly expressed in CRC and serves as an oncogene in CRC *via* promoting CRC cell oncogenesis and stem cell-like activity through the Wnt/β-catenin pathway (41, 42).

According to current research, the major function of YTHDF2 is to regulate mRNA degradation (43). Yang et al. found that YTHDF2 is upregulated in HCC, and miR145 is a negative posttranscriptional regulator of YTHDF2. Also, miR145 downregulates the expression of YTHDF2 through elevating m6A levels *via* binding to the 3′ UTR of YTHDF2 mRNA (14). On the contrary, Hou et al. revealed that YTHDF2 was downregulated in HCC. YTHDF2 deficiency robustly facilitated HCC growth and metastasis, suggesting that YTHDF2 is a tumor suppressor in HCC. Mechanistically, Hou et al. showed that YTHDF2 inhibited cancer progression by promoting the decay of IL11 and SERPINE2 mRNAs (44). Therefore, the specific role of YTHDF2 in HCC remains controversial. These contradictory findings may be due to the high heterogeneity of HCC. Further studies are required to address these conflicting observations.

Some studies have reported that deregulation of m6A regulators is associated with the drug resistance in tumors. Therefore, it is critical to know the expression of m6A regulators in cancer patients in order to choose individualized chemotherapeutic regimens. METTL3 is known to promote the resistance of pancreatic cancer cells against chemotherapy and radiotherapy. Knockdown of METTL3 can significantly increase the sensitivity of pancreatic cancer cells to 5-fluorouracil (5-FU), cisplatin, gemcitabine, and radiotherapy (45). Similarly, METTL3 is overexpressed in gliomas and is involved in the maintenance of its radio-resistance (31). In colorectal cancer, knockout of YTHDF1 can suppress the proliferation of cancer cells and enhance their sensitivity to chemotherapy drugs such as 5-FU and oxaliplatin (42). FTO is highly expressed in cervical squamous cell carcinoma (CSCC) tissues. By activating β-catenin and excision repair pathways, FTO enhances the chemo-radiotherapy resistance of CSCC (46). In tyrosine kinase inhibitor (TKI)-resistant leukemia cells, decreased m6A levels by FTO upregulation results in the overexpression of survival and proliferation-related genes. Vice versa, knockdown of FTO rendered resistant leukemia cells remarkably sensitive to TKI treatments (47). These studies highlight the significance of m6A in chemo-radiotherapy resistance and suggest its potential value as a treatment target. At present, there are no studies investigating the role of m6A in drug resistance in HCC. Nevertheless, given that METTL3 and YTHDF1 are significantly associated with prognosis of HCC patients, elucidating the mechanisms of m6A in HCC chemoresistance is of great importance for the treatment of drug resistance in HCC.

Previous studies have shown that m6A plays an important role in tumor initiation, progression, metastasis, and other malignant biological behaviors. Therefore, the development of specific inhibitors of m6A regulators has great scientific significance and clinical value. Rhein is the first FTO inhibitor that exerts its inhibitory effect by competitive binding to the FTO active site (48). However, rhein has the problem of low specificity due to its cross-activity with ALKB family demethylases (49). Meclofenamic acid (MA) is a highly selective FTO inhibitor that can bind to FTO and stabilize FTO without affecting the demethylase activity of ALKBH5 (50). In addition to natural products, Huang et al. developed two FTO inhibitors, namely, FB23 and FB23-2, by structure-based rational design. *In vitro* and *in vivo* experimental evidence demonstrated that FB23-2 exhibits a potent ability to suppress the progression of AML (51). More recently, based on structure-based virtual screening and a series of *in vivo* and *in vitro* experiments, Peng et al. discovered that entacapone, which was previously approved for the treatment of Parkinson's disease by the Food and Drug Administration (FDA), can be used as a specific inhibitor of FTO. Entacapone inhibits FTO activity through competitive binding with m6A-modified RNA substrates. After treatment with entacapone, the diet-induced obese mice showed a significant decrease in body weight and blood glucose levels (52). Science entacapone is already an FDA-approved drug, and it could be readily generalizable to other clinical indications that are related to FTO, such as cancer and obesity. For the time being, other than FTO, there are no known inhibitors for m6A regulatory. More effective and specific inhibitors for targeting m6A regulatory are urgently needed. The development of such inhibitors will not only deepen the understanding about the mechanism of m6A in carcinogenesis but also provide more tools for designing novel therapies.

Nevertheless, we acknowledge that several limitations in this study deserve mention. First, since our data are drawn from the TCGA and GEO databases, further experimental evidence is needed to verify our findings. Second, the sample size varied significantly between the normal and tumor groups, which may affect the reliability of the results. Finally, as the main patients are Americans and Italians, selection bias inevitably occurred. As a result, the findings in our study might not be generalizable to all populations.

In summary, we demonstrated that the gene expression signature of m6A modification regulators possesses great potential for HCC prognosis prediction. Our study offers additional evidence for further research regarding m6A RNA modification in HCC. However, further experimental and clinical exploration are necessary to confirm these findings.

## Data Availability Statement

All datasets presented in this study are included in the article/supplementary material.

## Author Contributions

SL, SZ, and LZha designed the study. LZha and YQ collected the data. JH, LZho, and DW performed the bioinformatics analysis. LZha and YQ wrote the manuscript. SL and SZ were responsible for the supervision of the work. All authors contributed to the article and approved the submitted version.

## Conflict of Interest

The authors declare that the research was conducted in the absence of any commercial or financial relationships that could be construed as a potential conflict of interest.
